# *Acanthamoeba *produces disseminated infection in locusts and traverses the locust blood-brain barrier to invade the central nervous system

**DOI:** 10.1186/1471-2180-10-186

**Published:** 2010-07-08

**Authors:** Parisa N Mortazavi, Graham Goldsworthy, Ruth Kirk, Naveed A Khan

**Affiliations:** 1 School of Biological and Chemical Sciences, Birkbeck, University of London, London, UK; 2 School of Life Sciences, Kingston University, Kingston-Upon-Thames, Surrey, UK; 3 School of Veterinary Medicine and Science, University of Nottingham, Sutton Bonington, UK

## Abstract

**Background:**

Many aspects of *Acanthamoeba *granulomatous encephalitis remain poorly understood, including host susceptibility and chronic colonization which represent important features of the spectrum of host-pathogen interactions. Previous studies have suggested locusts as a tractable model in which to study *Acanthamoeba *pathogenesis. Here we determined the mode of parasite invasion of the central nervous system (CNS).

**Results:**

Using *Acanthamoeba *isolates belonging to the T1 and T4 genotypes, the findings revealed that amoebae induced sickness behaviour in locusts, as evidenced by reduced faecal output and weight loss and, eventually, leading to 100% mortality. Significant degenerative changes of various tissues were observed by histological sectioning. Both isolates produced disseminated infection, with viable amoebae being recovered from various tissues. Histological examination of the CNS showed that *Acanthamoeba *invaded the locust CNS, and this is associated with disruption of the perineurium cell/glial cell complex, which constitutes the locust blood-brain barrier.

**Conclusions:**

This is the first study to demonstrate that *Acanthamoeba *invades locust brain by modulating the integrity of the insect's blood-brain barrier, a finding that is consistent with the human infection. These observations support the idea that locusts provide a tractable model to study *Acanthamoeba *encephalitis *in vivo*. In this way the locust model may generate potentially useful leads that can be tested subsequently in mammalian systems, thus replacing the use of vertebrates at an early stage, and reducing the numbers of mammals required overall.

## Background

*Acanthamoeba *is a multifaceted opportunistic pathogen that infects mainly immunocompromised people and/or contact lens wearers [1-4]. Despite advances in antimicrobial chemotherapy, the mortality rate associated with *Acanthamoeba *granulomatous encephalitis remains very high, i.e., > 90% [2,3,5]. This is, in part, due to our incomplete understanding of the pathogenesis and pathophysiology of *Acanthamoeba *encephalitis.

A whole-organism approach to the study of disease is considered essential in gaining a full understanding of the interrelationships between infectious agents and their hosts [6,7]. At present, mice are most widely used models to study *Acanthamoeba *granulomatous encephalitis *in vivo*. Mostly, *Acanthamoeba *granulomatous encephalitis is limited to individuals with a weakened immune system, so mice are pre-treated generally with corticosteroid to suppress the host defences, followed by intranasal inoculation of *Acanthamoeba *[8-11].

Although vertebrate model systems are seen as immediately more relevant, recent studies have demonstrated the possibility of using insects as a model to study *Acanthamoeba *pathogenesis *in vivo *[12]. Thus a major aim of this proposal is to generate wider acceptance of the model by establishing that it can be used to obtain important novel information of relevance to *Acanthamoeba *encephalitis without the use of vertebrate animals. Infection-induced anorexia [13,14] and locust mortality was determined for *Acanthamoeba *isolates belonging to the T1 and T4 genotypes. It is widely accepted that in the human infection due to *Acanthamoeba*, affected tissues other than the central nervous system (CNS) may include subcutaneous tissue, skin, liver, lungs, kidneys, adrenals, pancreas, prostate, lymph nodes and bone marrow, which suggests a haematogenous spread [5]. Here, histopathological specimens of infected locust tissues are used to determine whether *Acanthamoeba *produces disseminated infection in locusts. *In vitro *studies suggest that *Acanthamoeba *traverses the human blood-brain barrier by disrupting the human brain microvascular endothelial cells monolayers. Because the blood-brain barriers of insects comprise layers of cells joined by tight junctions, it is hypothesised that *Acanthamoeba *invades locust brains by modulating the integrity of the insect's blood-brain barrier.

## Results

### *Acanthamoeba *isolates belonging to genotypes T1 and T4 kill locusts

**Figure 1:**
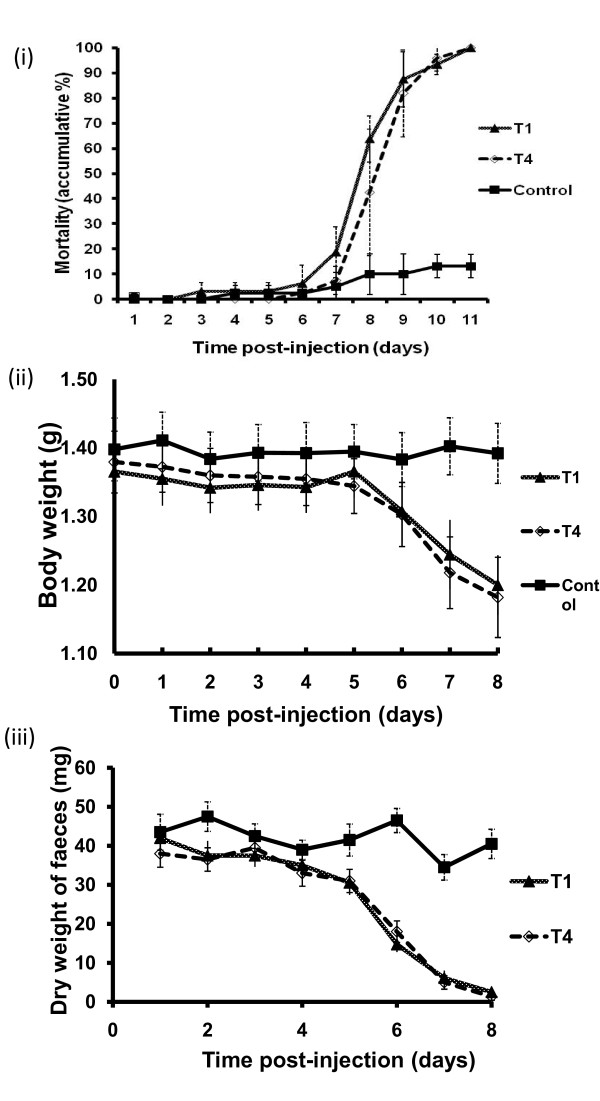
***Acanthamoeba *isolates belonging to the T1 and T4 genotypes induce sickness behaviour leading to locust death**. **(i) **Groups of 8 or 10 locusts (total n = 38 locusts/isolate) were injected with different isolates of *Acanthamoeba *(10^6 ^amoebae) and their mortality recorded every 24 h post injection. Mortality was 100% in all groups of amoebae-injected locusts within 11 days of infection, with the highest rate of death occurring between days 7-9. By contrast, locusts injected with culture medium alone, showed less than 15% mortality by day 11 post-injection. Results are representative of four independent experiments. **(ii & iii) **Groups of 6 or 7 locusts (total n = 20 locusts/isolate) were injected with different isolates of *Acanthamoeba *(10^6 ^amoebae) and their fresh weights recorded every 24 h post injection. Faecal pellets were also collected daily post-injection, air-dried and weighed. Both tested isolates of *Acanthamoeba *induced significant loss of body weight on day 8 (*P *< 0.05 using t-test; two sample unequal variance; one tail distribution) **(ii)**, as well as, faeces production (*P *< 0.05 using t-test; two sample unequal variance; one tail distribution) **(iii)**. Day 0 represents the injection day and error bars indicate S.E.M. of three independent experiments.

To determine whether *Acanthamoeba *isolates belonging to the T1 and T4 genotypes kill locusts, and if so, whether the speed of kill is similar among both genotypes, locusts in groups of 8 or 10 were injected with 10^6 ^amoebae of one of the isolates, and their mortality recorded every 24 h post injection. Both isolates of *Acanthamoeba *produced 100% mortality (Fig. 1i). More than 80% mortality occurred within 9 days of infection regardless of which genotype was tested, and this increased to 100% by day 11. The highest rates of mortality were observed between 7 - 9 days post-injection. Similar trends of mortality were observed in both groups of infected locusts, regardless of the amoeba isolate. By contrast, locusts injected with culture medium alone, showed less than 15% mortality by day 11 post-injection (Fig. 1i).

### *Acanthamoeba *isolates belonging to genotypes T1 and T4 induce anorexic effects in locusts

To quantify any possible anorectic effects in locusts due to *Acanthamoeba *injection, body weight changes and faeces production were monitored. Both isolates of *Acanthamoeba *induced sickness behaviour in infected locusts, as determined by significant loss of body weight on day 8 (*P *< 0.05 using t-test; two sample unequal variance; one tail distribution) (Fig. 1ii), as well as a reduction in faeces production (*P *< 0.05 using t-test; two sample unequal variance; one tail distribution) (Fig. 1iii). The reduction in body weight and faeces production of locusts was similar among all groups of locusts injected with different isolates of *Acanthamoeba *belonging to T1 and T4 genotypes. Of note, although locomotory behaviour was not quantified, after 5 days of infection locusts tended to be rather still and less excitable than non-infected locusts, often perching on a blade of wheat without attempting to eat.

### *Acanthamoeba *isolates of the T1 and T4 genotype each invade the locust brain

**Figure 2:**
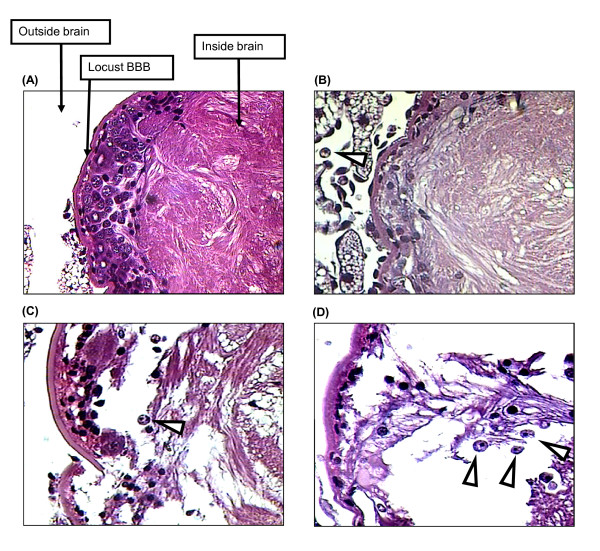
**Light micrographs of control-and *Acanthamoeba-*injected locust brains on different days post-infection**. Locusts were injected with 10^6 ^amoebae/culture medium only and their brains were isolated, fixed and sectioned on days 3, 5 and 7 post infection. Trophozoites of amoebae were observed inside the locusts' brains on days 5 **(C) **and 7 **(D) **post-infection, but not on day 3 **(B) **indicated by arrowheads. Disruption of the organisation within the brain tissue was also noticeable on days 5 and 7, but not on day 3. No amoeba or histopathological damage was observed in the control brains **(A) **and/or the capsule of the brain barrier. Note that the above images are representative micrographs of the genotype T4, but, similar results were observed with the T1 genotype. Magnification is × 400.

Brains of locusts injected with *Acanthamoeba *were dissected out and cultivated onto non-nutrient agar plates seeded with bacterial lawn. Amoebae were recovered from the brains of all groups of locusts injected with different *Acanthamoeba *isolates (data not shown). One hundred percent of amoebae-infected locusts showed the presence of amoebae in the brain lysates from day 5 onwards. As expected, lysates of non-infected control brains showed no growth of viable amoebae (data not shown). To further confirm the presence of amoebae within the CNS, brains from infected locusts were fixed, sectioned and stained using Harris' haematoxylin and eosin on days 3, 5 and 7 post-injection (three brains/isolate/day). Examination of the histological sections revealed that all amoebae isolates tested were able to invade the locust brain (Fig. 2). Trophozoites were observed inside locust brains on days 5 and 7, post-injection, but not on day 3 (Fig. 2). In general, few amoebae were found in the brains on day 5 post-injection (sometimes as few as 1 or 2 amoebae in the whole brain, but sometimes quite numerous), whereas on day 7 amoebae were always very numerous (data not shown).

### *Acanthamoeba *invades the locust brain by disrupting the blood-brain barrier

**Figure 3:**
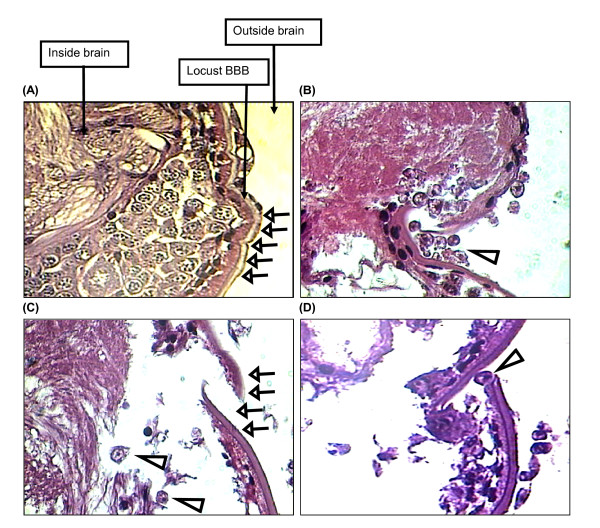
**Invasion of the locust brain by *Acanthamoeba *is associated with disruption of the outer capsule of the brain**. **(A) **Intact blood-brain barrier in control locusts (pointed by arrows). **(C) **Damaged blood-brain barrier of infected brain (pointed by arrows) with two amoebae inside the brain (indicated by arrowheads). **(B) **&**(D) **amoebae (indicated by arrowheads) appearing to penetrate the brain via broken blood-brain barrier. Note that the above images are representative micrographs of the genotype T4, but, similar results were observed with the T1 genotype. Magnification is × 400.

In histological sections of brains from infected locusts on days 5 and 7 after injection, amoebae were observed in most regions of the brain (Fig. 2 and 3). Notably, the presence of amoebae inside locust brains was associated often with clear evidence of a lesion in the brain capsule, especially on day 7 (Fig. 2). Furthermore, amoebae were observed in several cases (as illustrated in Fig. 2) in the vicinity of such lesions in the brain capsule, apparently in the process of invading the brain. Such lesions of the brain capsule were never observed in sections of brains from non-infected locusts, and were quite distinct from the occasional mechanical tears in tissue slices introduced during sectioning. In comparison with brains from control locusts, those from *Acanthamoeba*-infected locusts on days 5 and 7 showed gross disruption and degeneration of the internal organisation of the brain tissue, which was not seen on day 3 (Fig. 2). Isolates of both genotypes tested showed similar findings (data not shown). Moreover, amoebae entry into the locust brain was consistently observed with the breakdown of the blood-brain barrier, as shown in the representative images in Fig. 3). In controls, locusts' blood-brain barrier was always found to be intact (Fig. 3).

### *Acanthamoeba *isolates belonging to genotypes T1 and T4 disseminate within the locust body and invade various tissues

**Figure 4:**
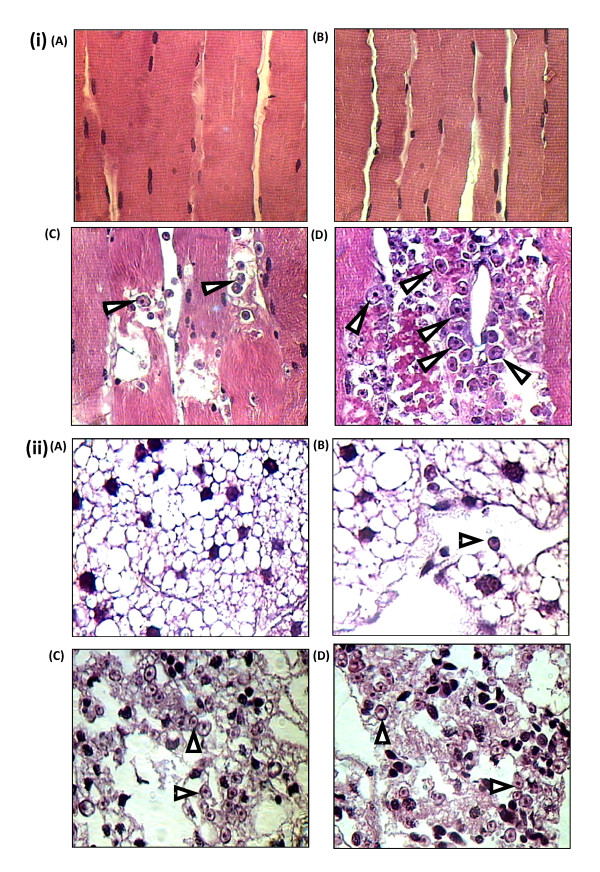
**Amoebae invade the locust's flight muscles as well as fat body surrounding the locust brain**. Dorsal-longitudinal flight muscle of *Acanthamoeba*-infected locusts were fixed in 4% paraformaldehyde and stained with Harris' haematoxylin and eosin. To determine infiltration of amoebae into the deep layers of muscle tissues, surface layers were removed and only deep tissues were sectioned. Trophozoites of amoebae (indicated by arrows) were detected in these tissue samples on days 5 **(C) **and 7 **(D) **post-infection, but not on day 3 **(B)**, nor in control muscles **(A)**. Degenerative changes of the tissues caused by the amoebae are significant on days 5 and 7. Note that the above images are representative micrographs of the genotype T4, but, similar results were observed with the T1 genotype. Magnification is × 400. **(ii) **Fat body surrounding the brain of *Acanthamoeba*-injected locusts was examined on days 3, 5 and 7 after injection. A large numbers of amoebae (pointed by arrowheads) were identified in the fat body on days 5 **(C) **and 7 **(D) **after injection. However, they were found in much fewer numbers on day 3 **(B)**. No amoeba was observed in the fat body of control locusts **(A)**. Note that the above images are representative micrographs of the genotype T4, but, similar results were observed with the T1 genotype. Magtnification is × 400.

Using plating assays, viable amoebae were recovered from the haemolymph of infected locusts on all tested days post injection (data not shown). Infected locusts showed the presence of numerous small black nodules in the head capsule and in the abdomen close to the point of injection (data not shown), suggesting that the locust's immune system had been activated by the presence of the amoebae [15,16]. Furthermore, trophozoites of amoebae were observed in large numbers in the histological sections of deep tissues of flight muscles on days 5 and 7 post-injection, but not on day 3. Degenerative changes in the tissues caused by the amoebae were apparent on days 5 and specifically 7 (Fig. 4i). Invasion of large numbers of amoebae into the fat body which was often surrounding the brain was evident in the histological studies on these days. Huge numbers of amoebae (both isolates) were identified in the fat body around the brains on days 5 and 7 after injection, but they were present in much lower numbers on day 3 (Fig. 4ii).

**Figure 5:**
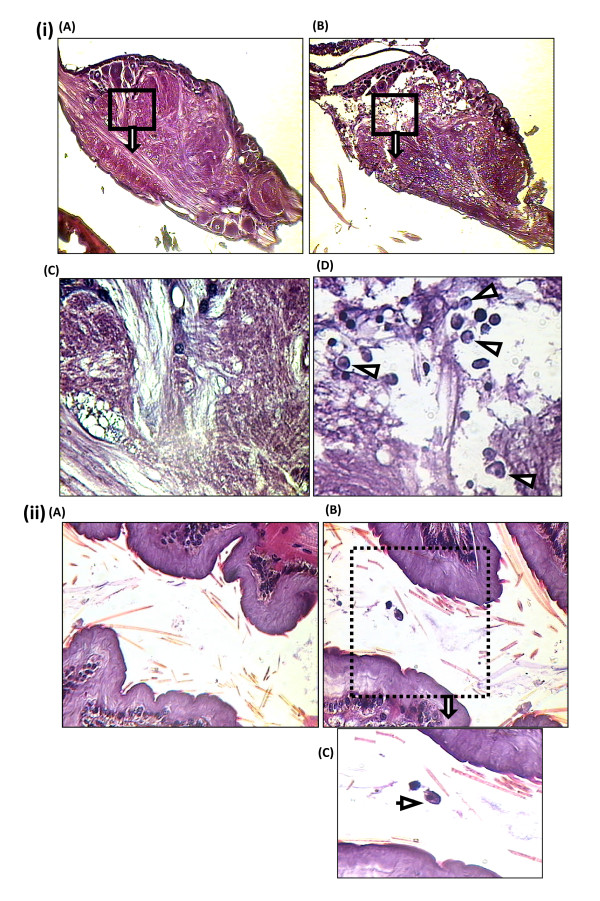
***Acanthamoeba *invades the locust suboesophageal ganglion and occasionally present in the lumen of the locust foregut**. **(i) (A) **Suboesophageal ganglia of control locust (×100); **(B) **Suboesophageal ganglia of infected locust (×100); **(C) **Suboesophageal ganglia of control locust at higher magnification (×400); **(D) **Suboesophageal ganglia of infected locust at higher magnification (×400). Note that the trophozoites of amoeba (indicated by arrowheads in **D**) were detected in the suboesophageal ganglion of infected locusts, but not in control locusts **(C)**. Histopathological damage in suboesophageal ganglia from infected locusts **(B) **was also evident, when compared with the ganglia in control locusts **(A)**. The above images are representative micrographs of the genotype T4, but, similar results were observed with the T1 genotype. **(ii) (A) **Foregut of control locust (×250); **(B) **Foregut of infected locust (×250); **(C) **Foregut of infected locust at higher magnification (×400). Trophozoites of amoeba (indicated by arrow in **C**) were identified in the lumen of the foregut of some of the locusts. No damage to the wall of the foregut was observed in the infected locusts. Note that the above images are representative micrographs of the genotype T4, but, similar results were observed with the T1 genotype.

Amoebae were observed not only in the brain of infected locusts, but also in the suboesophageal ganglion. Both isolates of *Acanthamoeba *infiltrated this ganglion and caused noticeable histopathological damage (Fig. 5i). Occasionally, sporadic amoebae in the form of trophozoites were seen also in the lumen of the foregut of some of the infected locusts (Fig. 5ii), but this was not a consistent finding in all foreguts sectioned.

## Discussion

The availability of *in vitro *models has made it possible to study, at the molecular level, *Acanthamoeba *interactions with the host cells, such as corneal epithelial/endothelial cells [17-20], brain microvascular endothelial cells [21], fibroblasts [22,23], keratocytes [18,22], macrophages [24-26], neutrophils [26,27], and neurones [28]. Although these models allow in-depth biochemical and molecular investigations *in vitro*, thus further elucidating mechanisms of infection, they cannot model whole organism responses to infection at the physiological level. This is particularly relevant in brain infection due to *Acanthamoeba *which involves complex interactions between amoeba and the host.

Both *Acanthamoeba *genotypes studied here in locusts, reduced faecal output at about 5 days post-injection, and killed all locusts within 11 days. Live *Acanthamoeba *can be recovered from brain lysates of amoebae-injected locusts, and trophozoites can be seen inside infected brains in histological studies. It is intriguing that amoebae are not found in the CNS of infected locusts on day three, and they invaded the brain after 4 or 5 days, with changes in faecal output and fresh body weight respectively becoming apparent. It is tempting to speculate from these temporal relationships that *Acanthamoeba*-mediated locust death is, at least in part, associated with the parasite's invasion of the brain. Interestingly, *Acanthamoeba *did invade other parts of the locust CNS such as the suboesophageal ganglion, but other ganglia (such as in the ventral nerve cord) were not investigated for the presence of amoebae in this study. The suboesophageal ganglion is situated below the crop and is connected to the brain by circumoesophageal connectives, and coordinates movements of the mouthparts, and the activity of the salivary glands. Clearly, invasion of the CNS by *Acanthamoeba *could affect feeding behaviour, as is suggested by the reduction in faecal output in infected locusts. It seems most likely that the changes in locust physiology and behaviour (reduction in body weight and faeces production, and reduced locomotory activity) are consequent on *Acanthamoeba*-mediated disruption of the blood brain barrier, which leads to neural dysfunction and reduced sensory output/input.

For the first time, histological examination of infected locusts shows that amoebae invaded deep into tissues such as the fat body and muscle, causing appreciable degenerative changes. Thus the amoebae invade these tissues, and are not isolated from them simply because they adhere to the surface of the tissues which are bathed in the haemolymph of the insect's open circulatory system. These findings suggest that *Acanthamoeba *produced parasitaemia and survived the onslaught of the innate immune defences of locusts. *Acanthamoeba *invaded different organs of the infected locusts and did not appear to exhibit any specific preference in relation to the tissues that are invaded, a finding that is consistent with the human form of infection, and confirms the validity of locusts as a useful model in which to study the pathogenesis of *Acanthamoeba *granulomatous encephalitis *in vivo*. Furthermore, *Acanthamoeba *granulomatous encephalitis is mostly limited to immunocompromised populations, and insects have an entirely innate immune defence system, suggesting that it is realistic to use locusts as a tractable model in which to study the pathogenesis of *Acanthamoeba *granulomatous encephalitis.

Although *Acanthamoeba *spread to many tissues and were found in the haemolymph throughout the course of the infection, none of the isolates (T1 and T4 genotype) were ever found in locust faeces (unpublished observations). For the first time, histological sectioning revealed the occasional presence of some amoebae in the lumen of the locust foregut, but no damage to the wall of the foregut was evident in any of the locusts subjected to microscopic examination. Indeed, the apical surfaces of the cells lining the foregut have a cuticle, which could represent a barrier to penetration by *Acanthamoeba*. Unfortunately, infected locusts destined for histological examination were not kept isolated from one another (as was the general case), and food replenishment and removal of dead animals took place only once every 24 h, so cannibalism was possible if locusts died shortly after this daily routine. It is likely therefore that amoebae observed occasionally in the lumen of foregut were simply there because they were consumed by cannibalism of a dead infected locust. This is a novel finding and it is strengthened by the fact that the histological sections never revealed evidence of damage to the wall of the foregut and suggest that amoebae do not infect locusts via the oral route, a finding that is consistent with infection in vertebrates.

Another significant finding was the entry of amoebae into the locust CNS, which appeared to be associated with disruption of the neural lamella and the perineurium/glial cell complex that constitutes the locust blood-brain barrier [29-31]. This is consistent with the studies *in vitro *showing that amoebae cross human brain microvascular endothelial cells, which constitute the blood-brain barrier, by affecting the integrity of the cell monolayer [32]. At present, the basis of the damage to the locust blood-brain barrier is not clear, i.e., amoeba and/or host inflammatory response. Recent studies *in vitro *show that serine proteases secreted by *Acanthamoeba *play an important role in affecting the integrity of the human brain microvascular endothelial cell monolayers [32], and the role of proteases and additional virulence determinants will be addressed in future studies *in vivo *using locusts. In addition, there is a need for a comparative study to test several additional *Acanthamoeba *isolates of various genotypes in locusts *versus *mice.

## Conclusions

The present findings suggest that locusts provide an excellent model to study the human *Acanthamoeba *granulomatous encephalitis and can elicit a full spectrum of host-pathogen interactions. The locust model can be a valuable tool to resolve the molecular and cellular features of *Acanthamoeba *granulomatous encephalitis and to determine the role of known as well as putative virulence determinants of *Acanthamoeba in vivo *that can be tested subsequently in mammalian systems. Such a technically convenient invertebrate model can be used for the initial screening and identification of novel virulence factors, providing useful leads for the rational development and evaluation of therapeutic interventions, and strengthen the move away from a total dependency on vertebrate models.

## Methods

### Locusts

Both male and female adult African migratory locusts (*Locusta migratoria*) between 15-30 days old were used as described previously [6,7]. Usually, experimental locusts were isolated individually in small (8 × 8 × 8 cm) wire-mesh cages in the insectary at 30°C throughout the course of the experiments, and fed daily with fresh grass and wheat seedlings supplemented with bran. Only in the histology experiments were injected locusts maintained together in groups of 10 in transparent plastic ‘critter cages' (28 × 17 × 17 cm, length × width × height). Notably, locusts are invertebrate pests and ethical approval is not required for their use in experiments.

### *Acanthamoeba *isolates and cultivation

Two clinical isolates of *Acanthamoeba *were used belonging to genotypes T1 (American Type Culture Collection, ATCC 50494; isolated from an *Acanthamoeba *encephalitis patient), and T4 (ATCC 50492; isolated from a keratitis patient). Based on the 18 S rRNA gene sequencing, most of the clinical isolates of *Acanthamoeba *(from keratitis, encephalitis and cutaneous infections) as well as environmental isolates have been typed as the T4 genotype, hence the aforementioned isolate was used as a representative of the T4 genotype. Amoebae were grown without shaking in 10 ml of PYG medium [0.75% (w/v) proteose peptone, 0.75% (w/v) yeast extract and 1.5% (w/v) glucose] in T-75 tissue culture flasks at 30°C as described previously [20,21] and media were refreshed 17 - 20 h prior to experiments. *Acanthamoeba *adherent to flasks represented trophozoite forms and were used for all subsequent assays.

### Mortality assays

To evaluate the virulence potential *in vivo*, mortality assays were performed as previously described [12]. Briefly, adult female locusts in groups of 8 or 10 (total n = 38 locusts for each isolate of amoeba) were injected with 10 μl of culture medium containing 10^6 ^amoebae. Suspensions of amoeba were injected into the haemocoel of a locust's abdomen through an intersegmental membrane between two abdominal terga. Control locusts were injected with the same volume of culture medium alone. Mortality of the experimental locusts was recorded every 24 h post-injection.

### Faecal production and changes in body weight

To determine the effects of *Acanthamoeba *on the ‘physiological well-being' of infected locusts, faeces production and changes in body fresh weight were monitored. Male locusts, in groups of 6 or 7, were injected with 10^6 ^amoebae in 10 μl of culture medium, and control locusts were injected with culture medium alone. To make the separation and collection of faeces of single locusts feasible, the experimental locusts were maintained in individual cages with a wire-mesh floor so that faecal pellets fell through and could be collected easily (and could not be eaten by the locusts, which are coprophagic). Whole body fresh weight of individual locusts was recorded at intervals of 24 h. At the same time, faecal pellets produced by individual locusts over the previous 24 h were collected, air-dried at room temperature overnight, and weighed.

### Parasitaemia and invasion of the CNS

To determine amoebic dissemination, samples of haemolymph (5 μl) were collected at 24-h intervals from day1 to 6 post injection, and inoculated onto non-nutrient agar plates seeded with *Escherichia coli *K-12 for the recovery of live amoebae. Plates were incubated at 30°C and examined daily under an inverted microscope. Haemolymph collection was performed as previously described [6,7]. Briefly, the cuticle and arthrodial membrane at the base of one hind leg of locust was sterilised with 70% ethanol, which was allowed to air-dry; the membrane was punctured using a sterile needle and the outflowing haemolymph was collected into 5 μl calibrated glass capillaries.

To determine whether different isolates of *Acanthamoeba *invaded the locust CNS, locust brains were isolated at 24 h intervals from day 1 to 6 post injection. To isolate the brains, the injected locusts were killed by decapitation, the left side of each head was removed by making a sagittal cut through the base of the left antenna, and each brain was dissected out. Each isolated brain was incubated with chlorhexidine (final concentration: 100 μM; Sigma Laboratories) at 37°C for 2 h to kill extracellular amoebae. Excess drug was removed subsequently by washing the brains with three separate 1 ml aliquots of PBS. Finally, the washed brains were disrupted physically using sterile pipette tips and by vigorous vortexing. These lysates were cultivated on bacteria-seeded agar plates. Plates were incubated at 30°C and the growth of *Acanthamoeba *was monitored daily using an inverted microscope.

### Histological studies

For histological studies, locusts were injected with 10^6 ^amoebae. On days 3, 5, and 7 post-injection, they were decapitated and their head capsules were fixed with 4% paraformaldehyde in PBS under vaccum for 72 h (a small cut was made in the frons to facilitate the collapse of the air sacs under vacuum and aid penetration of the fixative). The left and right sides of each fixed head were removed by making sagittal cuts through the bases of the left and right antennae; the brain with its circumoesophageal connectives to the suboesophageal ganglion intact, and still loosely attached to the foregut was dissected out using fine forceps: these preparations contained also some associated fat body tissue. The tissues were placed in fresh 4% paraformaldehyde in PBS for 48 h at room temperature. Fixed tissues were then dehydrated, cleared in Histo-Clear (National Diagnostics), infiltrated and embedded in Paramat (Gurr). Embedded tissues were sectioned at 5 μm using an automatic microtome; and the sections were stained with Harris' haematoxylin and eosin. Subsequently, sections were dehydrated, cleared in Histo-Clear and mounted in DPX resin (VWR BDH) under glass coverslips. Finally, slides were observed and photographed using a light microscope with a digital camera attached. Pieces of flight muscle tissue were also collected on the same days and fixed with 4% paraformaldehyde in PBS for 48 h at room temperature. To determine whether amoebae invaded deep tissues, surface layers of the fixed muscles were removed and the deep tissues were sectioned serially (5 μm thickness) as described above.

## Authors' contributions

NK conceived the study. PM and RK designed and performed the histological studies. PM, NK, and GG designed and performed all other assays. GG, PM, and NK did all statistical analyses on acquired data. NK and PM wrote the original manuscript. GG and RK helped to craft the final manuscript. All authors approved the final manuscript.

## References

[B1] Schuster FL Cultivation of pathogenic and opportunistic free-living amoebas Clin Microbiol Rev 2002 15 342 54 10.1128/CMR.15.3.342-354.2002 12097243 PMC118083

[B2] Schuster FL Visvesvara GS Free-living amoebae as opportunistic and non-opportunistic pathogens of humans and animals Int J Parasitol 2004 34 1001 27 10.1016/j.ijpara.2004.06.004 15313128

[B3] Marciano-Cabral F Cabral G *Acanthamoeba *Spp. as agents of disease in humans Clin Microbiol Rev 2003 16 273 307 10.1128/CMR.16.2.273-307.2003 12692099 PMC153146

[B4] Khan NA *Acanthamoeba *invasion of the central nervous system Int J Parasitol 2007 37 131 8 10.1016/j.ijpara.2006.11.010 17207487

[B5] Khan NA *Acanthamoeba *and the blood brain barrier: the breakthrough J Med Microbiol 2008 57 1051 7 10.1099/jmm.0.2008/000976-0 18719172

[B6] Khan NA Goldsworthy G Novel model to study virulence determinants of *Escherichia coli *K1 Infect Immun 2007 75 5735 9 10.1128/IAI.00740-07 17875634 PMC2168352

[B7] Mokri-Moayyed B Goldsworthy G Khan NA Development of a novel *ex vivo *insect model for studying virulence determinants of *Escherichia coli *K1 J Med Microbiol 2008 57 106 10 10.1099/jmm.0.47568-0 18065675

[B8] Culbertson CG Smith JW Cohen I Minner JR Experimental infection of mice and monkeys by *Acanthamoeba* Am J Pathol 1959 35 185 97 13617418 PMC1934806

[B9] Culbertson CG Ensminger PW Overton WM *Hartmannella *(*Acanthamoeba*), Experimental chronic, granulomatous brain infections produced by new isolates of low virulence Am J Clin Pathol 1966 46 305 14 5921408 10.1093/ajcp/46.3.305

[B10] Markowitz SM Sobieski T Martinez AJ Duma RJ Experimental *Acanthamoeba *infections in mice pretreated with methylprednisoloneor tetracycline Am J Pathol 1978 92 733 43 686155 PMC2018276

[B11] Mazur T Jozwiak M Extracerebral infections of *Acanthamoeba *spp. in mice Wiad Parazytol 1993 39 357 66 8128723

[B12] Mortazavi PN Goldsworthy G Kirk R Khan NA Novel model for the *in vivo *study of central nervous system infection due to *Acanthamoeba *spp. (T4 genotype) J Med Microbiol 2009 58 503 8 10.1099/jmm.0.005462-0 19273647

[B13] Exton MS Infection-induced anorexia: active host defence strategy Appetite 1997 29 369 83 10.1006/appe.1997.0116 9468766

[B14] Dantzer R Kelley KW Twenty years of research on cytokine-induced sickness behaviour Brain Behav Immun 2007 21 153 60 10.1016/j.bbi.2006.09.006 17088043 PMC1850954

[B15] Goldsworthy G Chandrakant S Opoku-Ware K Adipokinetic hormone enhances nodule formation and phenoloxidase activation in adult locusts injected with bacterial lipopolysaccharides J Insect Physiol 2003 49 795 803 10.1016/S0022-1910(03)00118-5 12880660

[B16] Wells KL The effects of immune challenge on phenoloxidase activity in locusts salivary glands *in vitro* Bioscience Horizons 2008 1 122 7 10.1093/biohorizons/hzn015

[B17] Morton LD McLaughlin GL Whiteley HE Effects of temperature, amebic strain, and carbohydrates on *Acanthamoeba *adherence to corneal epithelium *in vitro* Infect Immun 1991 59 3819 22 1894379 10.1128/iai.59.10.3819-3822.1991 PMC258957

[B18] Stopak SS Roat MI Nauheim RC Turgeon PW Sossi G Kowalski RP Thoft RA Growth of *Acanthamoeba *on human corneal epithelial cells and keratocytes *in vitro* Invest Ophthalmol Vis Sci 1991 32 354 9 1993587

[B19] Zheng X Uno T Goto T Zhang W Hill JM Ohashi Y Pathogenic *Acanthamoeba *induces apoptosis of human corneal epithelial cells Jpn J Ophthalmol 2004 48 23 9 10.1007/s10384-003-0018-y 14767646

[B20] Alsam S Jeong SR Dudley R Khan NA Role of human tear fluid in *Acanthamoeba *interactions with the human corneal epithelial cells Int J Med Microbiol 2008 298 329 36 10.1016/j.ijmm.2007.05.010 17931971

[B21] Alsam S Kim K Stins M Rivas AO Sissons J Khan NA *Acanthamoeba *interaction with human brain microvascular endothelial cells Microb Pathog 2003 35 235 41 10.1016/j.micpath.2003.07.001 14580387

[B22] Badenoch PR Adams M Coster DJ Corneal virulence, cytopathic effect on human keratocytes and genetic characterization of *Acanthamoeba* Int J Parasitol 1995 25 229 39 10.1016/0020-7519(94)00075-Y 7622330

[B23] Fritsche TR Sobek D Gautom RK Enhancement of *in vitro *cytopathogenicity by *Acanthamoeba *spp. following acquisition of bacterial endosymbionts FEMS Microbiol Lett 1998 166 231 6 10.1111/j.1574-6968.1998.tb13895.x 9770279

[B24] Stewart GL Kim I Shupe K Alizadeh H Silvany R McCulley JP Niederkorn JY Chemotactic response of macrophages to *Acanthamoeba castellanii *antigen and antibody-dependent macrophage-mediated killing of the parasite J Parasitol 1992 78 849 55 10.2307/3283316 1403427

[B25] Marciano-Cabral F Toney BM The interaction of *Acanthamoeba *spp. with activated macrophages and with macrophage cell lines J Eukaryot Microbiol 1998 45 452 8 10.1111/j.1550-7408.1998.tb05099.x 9703682

[B26] Hurt M Proy V Niederkorn JY Alizadeh H The interaction of *Acanthamoeba castellanii *cysts with macrophages and neutrophils J Parasitol 2003 89 565 72 10.1645/0022-3395(2003)089[0565:TIOACC]2.0.CO;2 12880257

[B27] Stewart GL Shupe K Kim I Alizadeh H Niederkorn JY Antibody-dependent neutrophil-mediated killing of *Acanthamoeba castellanii* Int J Parasitol 1994 24 739 42 10.1016/0020-7519(94)90129-5 7928077

[B28] Pettit DA Williamson J Cabral GA Marciano-Cabral F *In vitro *destruction of nerve cell cultures by *Acanthamoeba *spp.: a transmission and scanning electron microscopy study J Parasitol 1996 82 769 77 10.2307/3283890 8885887

[B29] Smith DS Treherne JE Functional aspects of the organization of the insect nervous system Adv Insect Physiol 1963 1 401 84 full_text

[B30] Treherne JE Schofield PK Mechanisms of ionic homeostasis in the central nervous system of an insect J Exp Biol 1981 95 61 73 7334320 10.1242/jeb.95.1.61

[B31] Carlson SD Juang JL Hilgers SL Garment MB Blood Barriers of the Insect Annu Rev Entomol 2000 45 151 74 10.1146/annurev.ento.45.1.151 10761574

[B32] Alsam S Sissons J Jayasekera S Khan NA Extracellular proteases of *Acanthamoeba castellanii *(encephalitis isolate belonging to T1 genotype) contribute to increased permeability in an *in vitro *model of the human blood-brain barrier J Infect 2005 51 150 6 10.1016/j.jinf.2004.09.001 16038767

